# ECOLOGICAL MOMENTARY ASSESSMENT OF ASSOCIATIONS AMONG HIGH AND LOW AROUSAL AFFECT AND COGNITIVE HEALTH

**DOI:** 10.1093/geroni/igz038.3491

**Published:** 2019-11-08

**Authors:** Eric S Cerino, Martin Sliwinski

**Affiliations:** 1 Pennsylvania State University, University Park, Pennsylvania, United States; 2 Penn State University, Center for Healthy Aging, University Park, Pennsylvania, United States

## Abstract

Negative affect (NA) and positive affect (PA) vary from moment-to-moment and these variations are associated with cognitive health. Past work has primarily focused on valence (negative/positive), however, largely ignoring the potential import of arousal (high/low). We address this gap by assessing the impact of high and low arousal NA and PA on daily cognition. A sample of 238 older adults (Mage=77.30 years, SD=5.14, Range=70–90) completed mobile surveys up to four times daily for 14 days. Participants reported current levels of high and low arousal NA and PA and performed processing speed and working memory tasks. For processing speed, there were significant within-person affect by age interactions. Moments when low arousal NA was higher than usual were associated with slower processing speed (Est.=0.87, SE=0.44, p<.05), and this effect was amplified in older participants (Est.=1.69, SE=0.60, p<.01). Moments when high arousal PA was higher than usual were associated with faster processing speed (Est.=-0.81, SE=0.40, p<.05), and this effect was amplified in younger participants (Est.=-1.81, SE=0.56, p<.01). For working memory, a significant within-person high arousal PA by age interaction emerged (Est.=0.001, SE=0.00, p=.046) such that moments when high arousal PA was higher than usual were marginally associated with worse working memory performance only among older participants (Est.=0.004, SE=0.002, p=.06). Results suggest momentary increases in low arousal NA and high arousal PA may confer greatest risk to daily cognitive health among older adults with more limited capacity and/or cognitive resources, whereas affective influences may be more facilitative among comparatively younger adults.

